# Decomposition of Ethiopian life expectancy by age and cause of mortality; 1990-2015

**DOI:** 10.1371/journal.pone.0204395

**Published:** 2018-10-03

**Authors:** Gizachew Balew Jembere, Youngtae Cho, Myunggu Jung

**Affiliations:** 1 Graduate School of Public Health, Seoul National University, Seoul, Korea; 2 Faculty of Epidemiology and Population Health, London School of Hygiene & Tropical Medicine, London, United Kingdom; Leibniz Institute for Prevention Research and Epidemiology BIPS, GERMANY

## Abstract

Ethiopia’s average life expectancy has improved by more than 18 years from 1990 to 2015. This initiated interest to study the gain in life expectancy with respect to age structure and cause of death. Applying a life expectancy decomposition technique on secondary data obtained from the Institute of Health Metrics and Evaluation, the study found that the burden of disease in Ethiopia has declined from 626.18 in 1990 to 225.69 in 2015 per 1000 population measured in age-standardized rate of life years lost. The major causes of burden in 1990; namely lower respiratory tract infections, neonatal disorders, diarrheal diseases and neglected tropical diseases at rates of 89.2, 63.2, 61.2, and 42.2 age-standardized years of life lost per 1000 population respectively; have shown a fast decline in 2015. Deaths from neglected tropical disease showed 94.95% reduction, contributing to 5.71(27.30%) years gain in life expectancy followed by lower respiratory tract infection and diarrheal disease contributing about 4.65 years (22.23%) and 1.48 years (7.10%) respectively. On the other hand, about 3.3 (15.73%) years and 6.4 (30.71%) years of increase in life expectancy are achieved through improved longevity in infants and children aged 1–4 years respectively. In conclusion, the study found that reductions in under-five child mortality and decline in burden of major communicable diseases could explain the major gain in life expectancy. However, findings also revealed that the prevalence of non-communicable diseases and injuries are on the rise calling for the need to be addressed by the public health system.

## Background

Life expectancy has been utilized as a quantitative measure of mortality and longevity within and across societies. According to Raza et.al., the last decade in the 20^th^ century was characterized by stagnation in life expectancy owing to an increase in morbidity and mortality from Human Immuno-Virus/Acquired Immuno-Deficiency Syndrome (HIV/AIDS); especially in sub-Saharan Africa [1]. Despite this challenge, the 2014 World Health Organization (WHO) report shows that globally a girl who was born in 2012 can expect to live for about 73 years, and a boy to the age of 68, which is about six years above the 1990 average [2,3]. According to these reports, seven sub-Saharan African countries have gained a life expectancy of more than 10 years; Liberia and Ethiopia being on top of the list by gaining a total of 20 and 19 years from 1990 to 2010 [2–5], respectively. The result from the 2016 Global Burden of Disease (GBD) study indicates that, globally disability adjusted life years (DALYs) of communicable diseases has decreased from 211.63 to 90.34 per 1000 (57.31%). Similarly, in Ethiopia, communicable diseases have decreased from 820.29 per 1000 to 200.87 per 1000 (75.51%). In 2013, diarrheal and lower respiratory tract infections have reduced from 67 to 19 and from 22 to 7.6 per thousand, respectively. Non-communicable diseases on the other hand have increased from 196.07 to 140.21 per 1000 (28.49%) in Ethiopia.

Ethiopia, a country with over 102 million population and a total fertility rate of 4.6, is one of the least developed countries that has recently registered an improvement in the human development index [6,7]. The public health sector is one of the areas where improved progress is seen across time in the country. The number of health centers has increased from about 600 in 1990 to 2999 in 2011, and previously non-existent health extension workers of 35,000 are actively working in the community. However, the health service utilization estimated by the outpatient service consumption is still very low, showing an increase from 0.2 to 0.3 from 1990 to 2011 [8,9] with a high rural urban gap. The 2016 demographic and health survey on the other hand indicated an improvement in the proportion of underweight children to 23 percent, down from 47.6 percent in 1990. Similarly, under-five mortality has reduced from 184 to 67 per 1000 children and infant mortality from 111 to 48 per 1000 live born infants [7,9,10]. Generally there is an increasing evidence supporting the improvement in children’s health conditions in Ethiopia; supported by the achievement of a two thirds reduction in child mortality, two years ahead of the millennium development goal time line [11].

Living in a resource scarce world; health sector planning, resource allocation and implementation requires an understanding of the major causes of morbidity and mortality that affect longevity and productivity at the community level. In this context, understanding the contributions of the age and cause specific factors on the improvement in life expectancy will help for further national and regional planning and resource allocation, despite data limitations that pose a huge bottleneck in most developing countries. Nonetheless, after the introduction of DALY in 1993 [12,13] there have been various small scale and large scale studies that decompose mortality by age and cause to help understand premature mortality, disability adjusted life years, life years lost, and similar other measurements of disease burden. On the other hand, in spite of the increasing interest in understanding life expectancy and the availability of various methods to decompose it, there are few researches conducted to understand the dynamics of life expectancy across communities. This gets much worse for low-income countries, where adequate data and especially vital registrations are almost non-existent to understand the age and cause specific morbidity and mortality in a community.

Thanks to the global burden of disease study from the world health organization and the Institute of Health Metrics and Evaluation (IHME), estimates of such cause specific mortality for every country are available online[13]. The main objective of this study is to analyze the gain in life expectancy in Ethiopia from 1990 to 2015. More specifically, life gained in the past two and half decades will be decomposed to investigate the age and cause specific contributions in the improvement of life expectancy in the county.

## Methods

This research is a quantitative study of life expectancy decomposition across time between abridged age intervals and among different causes of mortality using secondary data for Ethiopia. The data is retrieved from the Global Burden of Disease Collaborative Network, the 2016 Global Burden of Disease Study available at the IHME website

### Data source and data management

The global burden of disease study provides a comprehensive data to assess national, regional and global trends in age-specific, sex-specific, and cause-specific mortality. Estimation of these data depends on vital registration in countries where such data is available [14,15]. In the case of developing nations, vital registration is either incomplete or nonexistent; in which case census data, demographic data, health survey, and verbal autopsy literature reviews help to estimate burden of diseases. For many countries where data were missing for some years, both interpolation and extrapolation techniques have been applied to estimate mortality rates. For high-mortality countries with under-five mortality ratio of above 35 per 1000 live births in 2000–10 and without adequate death registration, data on causes of under-five deaths was estimated using a verbal-autopsy-data-based multi-cause statistical model (VAMCM) [14,15]. Specifically, under-five mortality in Ethiopia is estimated using data from census (1984, 1994, 2007), demographic health surveys (2000, 2005, 2011), Butajira in-depth surveys, and national family and fertility surveys (1990) [14,15]. According to the GBD protocol, a cause list is a set of mutually exclusive and collectively exhaustive hierarchical categories where the sum of each cause-specific mortality equals all-cause mortality, following international classification of disease. The revised list of GHE2015 cause is built out of newly added causes of death from the 2013 causes, together with corresponding 10^th^ version of the International Classification of Disease (ICD-10) codes that contains 333 causes [16–18]; whereas the GBD 1990 cause list used the ICD 9/10 classification. With a thorough understanding of the strong methodological approach for disease estimation that the GBD study uses, data was downloaded directly from its website.

The data retrieved from IHME site is presented in different hierarchical levels; level I includes communicable diseases, maternal causes, perinatal conditions, and nutritional deficiencies. level II includes non-communicable diseases, while level III includes intentional and unintentional injuries. This data is available in the form of the number of deaths all in point estimates and 95% uncertainty intervals. This study uses point estimate data on the number of deaths for both sexes in 1990,2000, 2010 and 2015. The team conducted data exploration and arrangement for further analysis using the ICD-10 list of causes of mortality under hierarchy II and hierarchy III. Among all lists of cause of mortality, based on a descending order, the top 19 causes of mortality were filtered out and included in this duty. The rest of the list of causes of mortality were merged in to the “Others” category. A final list of 20 causes of mortality including other causes were merged with the corresponding population age structure extracted from the UN population data released in the 2015 projection [19]. This data file was then arranged in text format to match the requirements of the survival six program, a DOS application used by demographers for survival analysis and life table constructions.

### Data analysis

Different scholars across the globe had developed a number of statistical methods to analyze life expectancy variations and their underlying causes. These include Pollard JH (1982, 1988), Arriaga EE (1984), Pressat R (1985), Andreev EM (1982) and Keyfitz N (1977, 1985) who are all pioneers in the field of demography [20]. Earlier studies indicate that Pollard's method is an exact decomposition of life expectancy, whereas Arriaga’s method is slightly easier to calculate while being more of an approximate method. However, comparisons of these methods have shown that the results obtained in either of the methods are comparable, especially for Arriaga’s and Pollard’s method [21,22]. After importing the data in to the survival-6 software program, a multi decrement abridged life table was constructed. Using the life expectancy output from the survival program, further decomposition of the gain in life expectancy from 1990 to 2015 was done using Pollard’s method in excel 2013 [20,21]:

### Pollard’s life expectancy decomposition formula

$$life\;\exp\;\left(diff\right)=e_{01}-e_{02}=\Sigma \left({\text{m}}_{\text{x}1}-m_{x2}\right)*w$$

$$\text{w}=\frac{\text{n}}{2}*\left(\left(\frac{l_{x1}e_{x2}+l_{x2}e_{x1}}{2}\right)+\left(\frac{l_{x+n1}e_{x+n2}+l_{x+n2}e_{x+n1}}{2}\right)\right)$$

The two expressions in parentheses above; the right and left ones, indicate the weights at times t_i_ and t_i+n_ respectively. To decompose the last open group—age 80+, the formula is replaced with:

$${\text{e}}_{01}-{\text{e}}_{02}=\Sigma \left({\text{m}}_{\text{x}1}-{\text{m}}_{\text{x}2}\right)\text{*}\frac{1}{2}\text{*}\left(\frac{{\text{T}}_{\text{x}1+}}{{\text{m}}_{\text{x}2+}}+\frac{{\text{T}}_{\text{x}2+}}{{\text{m}}_{\text{x}1+}}\right)$$

Keys:

n = age interval (five years commonly)

e_01_ = Life expectancy at age 0 in population one

e_02_ = Life expectancy at age 0 in population two

e_x+n1_ = Life expectancy after age “x+n” in population one

e_x+n2_ = life expectancy after age “x+n” in population two

l_x1_ = number surviving to the beginning of the age (x) Interval in population one

l_x2_ = number surviving to the beginning of the age (x) interval in population two

l_x1+n1_ = Number surviving at n years after age “x” in population one

l_x2+n2_ = Number surviving at n years after age “x” in population two

w = weight

m_x1_ = mortality at age “x” in population one

m_x2_ = _mortality at age “x” in population two

T_x_ = Total person years of life contributed after attaining age “x”

## Results

**Fig 1:**
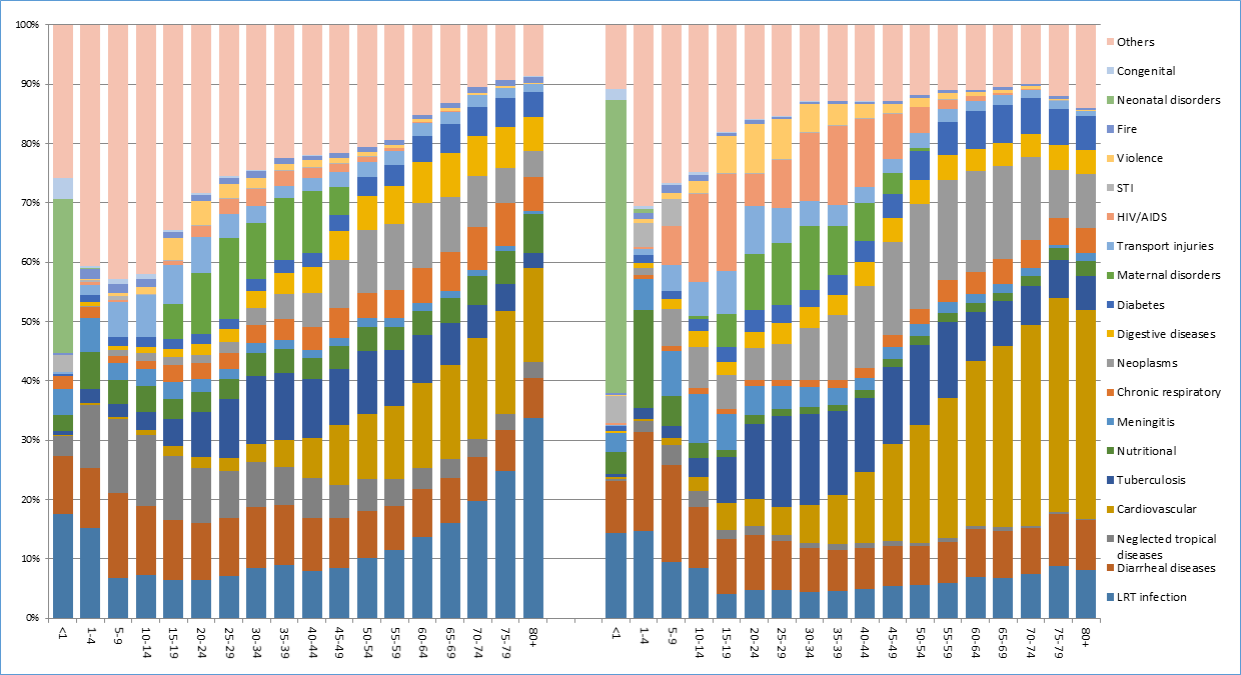
The distribution of age-cause specific proportion of deaths across abridged age groups in Ethiopia, 1990, 2015. This figure illustrates the mortality proportion for each specific cause of disease across a five year abridged age category in the horizontal axis, except for age under one and under five where age intervals are one year and four year respectively. The left graph indicate the data for 1990 and the right one indicate the data for 2015 estimate based on the global burden of disease study.

The top 19 causes of mortality and morbidity were included in the model to study life expectancy differences. A simple age specific mortality ratio shows that these causes explain about 65% of under one mortality, an estimated 58% under-five mortality, and more than 80% of mortality above the age of 60 in the year 1990. For the 2015 data, the above causes of mortality explained more than 90% of infant mortality and 80% of mortality above age 35 (Fig 1). This figure shows that mortality from cardiovascular diseases, lower respiratory tract infection, neglected tropical diseases, diarrheal diseases, neoplasms and tuberculosis are the major causes of death in 2015 with a slight shift in their order compared to those in 1990.

Further analysis was conducted on the burden of disease from a multi decrement life table (Table 1) using the mean YLL for each specific cause and year; and the percentage reduction in years of life lost after age standardization. Mean years of life lost quantifies the average year that a person would have lived had he or she not died prematurely from a cause. A higher value indicates a premature death and a lower value indicates an improved longevity. It is calculated as the sum of individual years of life lost at each age category using life expectancy as age limit in each age category.

$$YLL=\Sigma d_{it}*e_{t},$$

Where,

d_it_ = number of deaths at a given age category

e_t_ = standard life expectancy at age of death in years for each cause

However, for the sake of comparison YLL is weighted based on age specific reference population that is taken to be the average of the populations in 1990 and 2015. There are two mechanisms for comparing years of life lost across populations. One of these methods uses the rate of years of life lost and the second involves age standardization of years of life lost for each cause [23]. The following method of calculating the rate of YLL helps to compare the burden of disease across different populations or across different time points [23].

$$rate\;YPLL=\Sigma e_{t}d_{it}*1000/N$$

Where N = Total population for that country in that year.

Our calculation for this study shows that the rate of YLL in 1990 and 2015 are 684.8 and 218.9 per 1000, respectively. This indicates a higher burden of disease in 1990. This finding also matches the findings of Abdulahi et.al. [24]; which shows burden of disease to be 59,125 per 100,000 using DALYs. Further comparison of YLL across time and between different populations for specific causes of mortality requires standardization of years of life lost to eliminate the effect of different age structures among the different populations, usually with direct method of standardization [23]. In this study, age-standardized rate of YLL is estimated as follows:

$$age\;standardized\;YLL=\frac{\Sigma e_{t}d_{it}}{Pi}*(\frac{P_{ir}}{N_{r}}*1000)$$

Where;

d_it_ = deaths for each specific cause of mortality by abridged age category

e_t_ = life expectancy by abridged age category

P_i_ = number of persons of age *i* in the actual population

P_ir_. = number of persons of age *i* in the reference population

N_r_ = Total number of persons in the abridged age category in the reference population (total population)

**Table 1:** Age-standardized rate of years of life lost in Ethiopia, from 1990 to 2015.

Cause of death	Age-standardized mean YLL				Diff (1990–2015)	Reduc. (%)
	1990	2000	2010	2015		
LRT infection (CD)	89.22	47.96	39.47	22.69	66.53	16.61%
Diarrheal diseases (CD)	61.19	41.61	22.71	21.63	39.56	9.88%
Neglected tropical diseases (CD)	42.16	12.47	25.03	2.13	40.03	9.99%
Cardiovascular/Circulatory dis. (NCD)	14.12	20.64	12.10	17.62	-3.49	-0.87%
Tuberculosis (CD)	22.68	26.32	13.04	13.03	9.64	2.41%
Nutritional deficiencies (CD)	25.44	17.02	13.74	9.63	15.81	3.95%
Meningitis (CD)	24.26	12.67	11.73	7.95	16.30	4.07%
Chronic respiratory dis. (NCD)	15.76	3.45	7.40	2.69	13.08	3.27%
Neoplasms (NCD)	10.45	12.24	10.80	13.36	-2.91	-0.73%
Digestive diseases (NCD)	8.79	5.84	6.12	4.51	4.28	1.07%
Diabetes, (NCD)	8.50	6.68	6.55	5.76	2.74	0.68%
Maternal disorders (CD)	12.09	10.85	4.99	4.79	7.30	1.82%
Transport injuries (INJ)	11.59	6.16	9.74	4.87	6.73	1.68%
HIV/AIDS (CD)	3.81	32.95	10.69	8.69	-4.88	-1.22%
STI (CD)	7.63	6.36	4.32	5.31	2.32	0.58%
Interpersonal violence (INJ)	3.95	3.63	4.52	3.74	0.21	0.05%
Fire, heat, and hot substances (INJ)	5.79	1.77	3.80	1.09	4.70	1.17%
Neonatal Disorders (CD)	63.18	51.38	43.57	40.16	23.03	5.75%
Congenital birth defects (CD)	9.98	2.62	3.77	1.83	8.16	2.04%
Others	185.60	115.05	54.02	34.23	151.36	37.79%
**Total**	626.18	437.67	308.12	225.69	400.50	
**Entropy**	0.48	0.37	0.28	0.23		

This table shows the age-standardized mean years of life lost (mYLL), column two to five for 1990, 2000, 2010 and 2015 respectively. The sixth column indicates the difference in rate of age-standardized years of life lost from 1990 to 2015 while the last column indicates the percentage change. (CD: Communicable, maternal, neonatal, and nutritional diseases, NCD: Communicable, maternal, neonatal, and nutritional diseases, INJ: Injuries)

**Fig 2:**
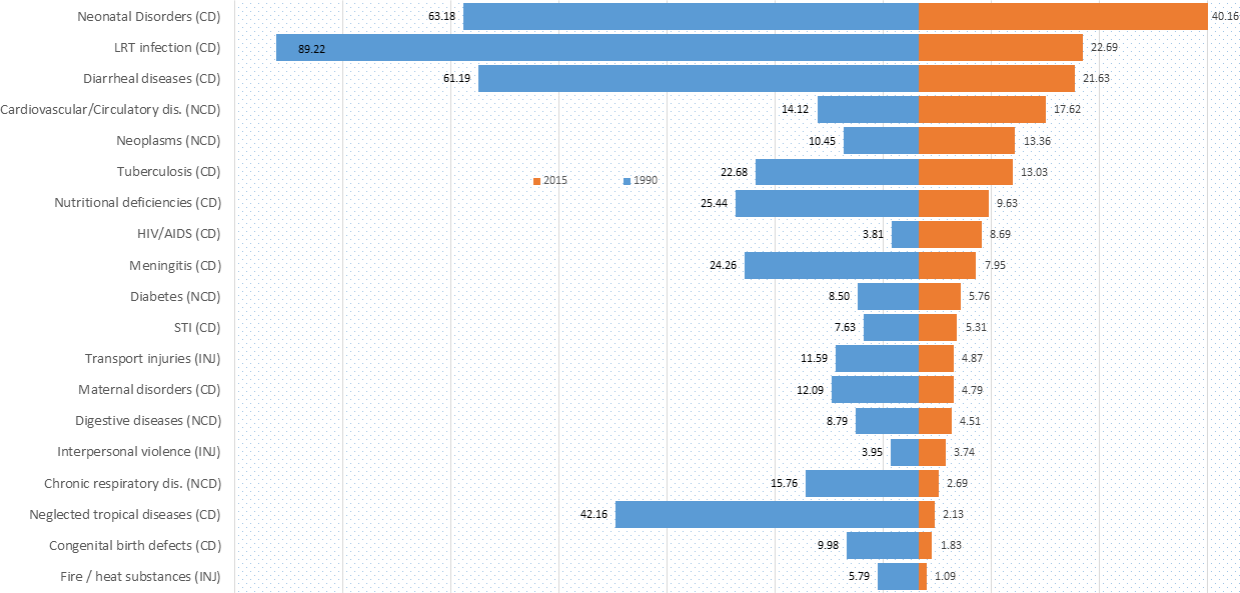
Age-standardized years of life lost by cause for 1990 and 2015 in Ethiopia. This figure indicates the age-standardized years of life lost for 1990 and 2015 in Ethiopia. The list in the y-axis indicates the top 19 lists of cause of death whereas, the values on the x-axis indicate the life years lost per 1000 standard population.

The findings of years of life lost calculations in this study (Table 1 and Fig 2) indicate that lower respiratory tract infections (LRT), neonatal disorders, diarrheal disease, and neglected tropical diseases, are the top causes of premature death in 1990, evidenced by their order of age-standardized mean YLL; 89.2, 63.2, 61.2 and 42.2 per 1000 persons respectively. In 2015, a shift in order is noticed with; neonatal disorders, LRTI, diarrheal diseases, neglected tropical diseases and cardiovascular diseases being the top causes of premature death at a value of 40.2, 22.7, 21.6 and 17.6 per 1000 persons respectively. A reduction in years of life lost could be either due to a reduction in number of deaths or an upward shift in age of death. This is in line with the goal of public health; reduction of mortality and elongation of life.

The reduction in the rate of YLL calculated based on the age-standardized deaths shows that most causes of death have registered a fall in rate of years of life lost from 1990 to 2015. Along with a reduction in rates of years of life lost by 66.5, 40.2, 40.0, and 39.6 per 1000 persons, deaths from conditions such as lower respiratory tract infections, neonatal disorders, neglected tropical diseases and diarrheal diseases respectively showed the highest fall from 1990 to 2015. Based on the percentage reduction, deaths from lower respiratory infections showed a 16.6% reduction from 1990 to 2015. Similarly, deaths from neglected tropical diseases and diarrheal diseases decreased by 10% and 9.9% respectively. On the contrary, non-communicable diseases including cardiovascular diseases and neoplasms show an increase in burden of disease by almost 1% for both of the causes.

Another method of evaluation of burden of disease was conducted using the cause elimination life table construction. Based on this method, life expectancy before cause-elimination and after cause-elimination is calculated. The difference in life expectancy is expected to be the potential life expectancy that will be gained if we eliminate that cause. Table 2 shows the potential life years gained after cause elimination. Column 1 contains causes of mortality while Column 2 5 show potential life years gained from 1990 to 2015 for each specific cause respectively. The higher the potential gain, the higher the burden of that disease in the community. The findings illustrate that the order of burden of disease matches the burdens measured using rate of years of life lost with slight changes in rankings. Most communicable diseases showed steady declines in potential life years gained, including lower respiratory tract infections (4.2 to 1.2), diarrheal diseases (3.1 to 1.3), neglected tropical diseases (2.3 to 0.1) and tuberculosis (2.0 to 1.4) through the four periods. However, non-communicable disease endured slightly different trends. Despite a decreasing burden by digestive disease (Table 2), potential life years gained by cardiovascular diseases and neoplasms increased in 2015 in comparison with its figures in 1990.

**Table 2:** Potential life years gained by cause elimination, 1990 to 2015 in Ethiopia.

Cause of Mortality	1990	2000	2010	2015
**LRT infection (CD)**	4.20	2.07	2.19	1.17
**Diarrheal diseases (CD)**	3.06	2.24	1.06	1.34
**Neglected tropical diseases (CD)**	2.25	0.66	1.39	0.14
**Cardiovascular and circulatory diseases (NCD)**	1.89	2.96	1.71	2.58
**Tuberculosis (CD)**	2.00	2.83	1.30	1.42
**Nutritional deficiencies (CD)**	1.45	0.83	0.89	0.46
**Meningitis (CD)**	0.99	0.63	0.52	0.42
**Chronic respiratory diseases (NCD)**	1.17	0.48	0.68	0.38
**Neoplasms (NCD)**	1.42	1.67	1.46	1.78
**Digestive diseases (NCD)**	1.05	0.64	0.78	0.51
**Diabetes, (NCD)**	0.77	0.73	0.69	0.67
**Maternal disorders (CD)**	0.93	0.88	0.37	0.38
**Transport injuries (INJ)**	0.78	0.48	0.72	0.40
**HIV/AIDS (CD)**	0.28	2.84	0.77	0.67
**STI (CD)**	0.17	0.17	0.09	0.13
**Interpersonal violence (INJ)**	0.27	0.27	0.31	0.28
**Fire, heat, and hot substances (INJ)**	0.34	0.12	0.23	0.08
**Neonatal Disorders (CD)**	1.16	0.90	0.71	0.65
**Congenital birth defects (CD)**	0.22	0.06	0.09	0.04
**Others**	8.60	5.93	3.01	2.15
**Total**	32.95	27.40	18.95	15.61
**Life expectancy to age 0**	47.05	52.60	61.05	64.39

This table shows the life years gained by each specific cause of mortality calculated using the difference in life expectancy before cause elimination and after cause elimination. This value is expected to be the life expectancy that will be gained if we eliminate that specific cause. Column one contains causes of mortality while Column 2 to 5 show life years gained for the year 1990,2000, 2010 and 2015 for each specific cause.

Improvement in life expectancy from a specific cause of death may result either through an upward shift in age specific mortality or through a reduction in the incidence of diseases. This necessitates the application of the decomposition method to study the gain in life expectancy that can be apportioned to either population age structure or cause of death. Table 3 shows the contribution of age structure on gains in life expectancy in Ethiopia from 1990 to 2015, calculated using the age and cause decomposition of life expectancy. Column one indicates age interval, column 2 to 5 indicate expected life expectancy in 1990, 2000, 2010 and 2015. Column 6 compares age-specific life expectancy between 1990 and 2015. Column 7 indicates differences in life expectancy attributed to specific age interval and the last column represents the percent share of this difference to the total gain in life expectancy. The values in the result demonstrate that a gradual increase in life expectancy at birth by about 18.3 years, from 47.7 in 1990 to 66.00 in 2015. It is important to note that the decomposition analysis indicates the difference across time to be 20.89, which is due to residual effects. Observing across age structure, among the total 18.3 years increase in life expectancy from 1990 to 2015, about 6.4 (30.71%) years of the total increase in life expectancy is achieved through improved longevity in children ages 1–4 years of old. Another 15.7% improvement of years in longevity is achieved by mortality reduction in infants (Table 3). Altogether, reduction in under-five mortality makes up more than 45.8% of the gain in life expectancy.

**Table 3:** The contribution of age structure for the difference in life expectancy from 1990 to 2015.

Age	1990	2000	2010	2015	Attribution (1990 vs 2015)	Percentage (%)
**<1**	47.74	53.16	62.99	66.00	**3.29**	15.73
**1–4**	50.45	54.31	63.73	66.22	**6.42**	30.71
**5–9**	53.58	54.21	61.70	63.37	**0.42**	2.00
**10–14**	49.22	49.83	57.01	58.62	**0.34**	1.61
**15–19**	44.77	45.28	52.26	53.86	**0.46**	2.20
**20–24**	40.58	40.96	47.75	49.25	**0.75**	3.61
**25–29**	36.79	37.01	43.48	44.73	**0.82**	3.90
**30–34**	33.13	33.29	39.27	40.28	**0.73**	3.51
**35–39**	29.48	29.73	35.15	35.97	**0.86**	4.14
**40–44**	26.14	26.29	31.22	31.72	**0.69**	3.32
**45–49**	22.74	22.91	27.37	27.64	**0.67**	3.21
**50–54**	19.49	19.61	23.71	23.72	**0.71**	3.42
**55–59**	16.54	16.46	20.20	20.03	**0.58**	2.76
**60–64**	13.67	13.50	16.94	16.54	**0.75**	3.59
**65–69**	11.50	10.80	14.06	13.36	**0.49**	2.36
**70–74**	9.48	8.46	11.63	10.54	**0.35**	1.69
**75–79**	7.94	6.58	9.81	8.18	**0.10**	0.47
**80+**	6.47	5.05	8.28	6.29	**2.46**	11.77
**Total**					**20.89**	100

Table three shows the contribution of age structure on gains in life expectancy in Ethiopia from 1990 to 2015, calculated using the age and cause decomposition of life expectancy. Column one indicates age interval, column 2 to 5 indicate life expectancy in 1990,2000, 2010 and 2015 respectively, column six indicate difference in life expectancy attributed to specific age interval from 1990 to 2015 and the last column represents the percent share of this difference to the total gain in life expectancy.

**Fig 3:**
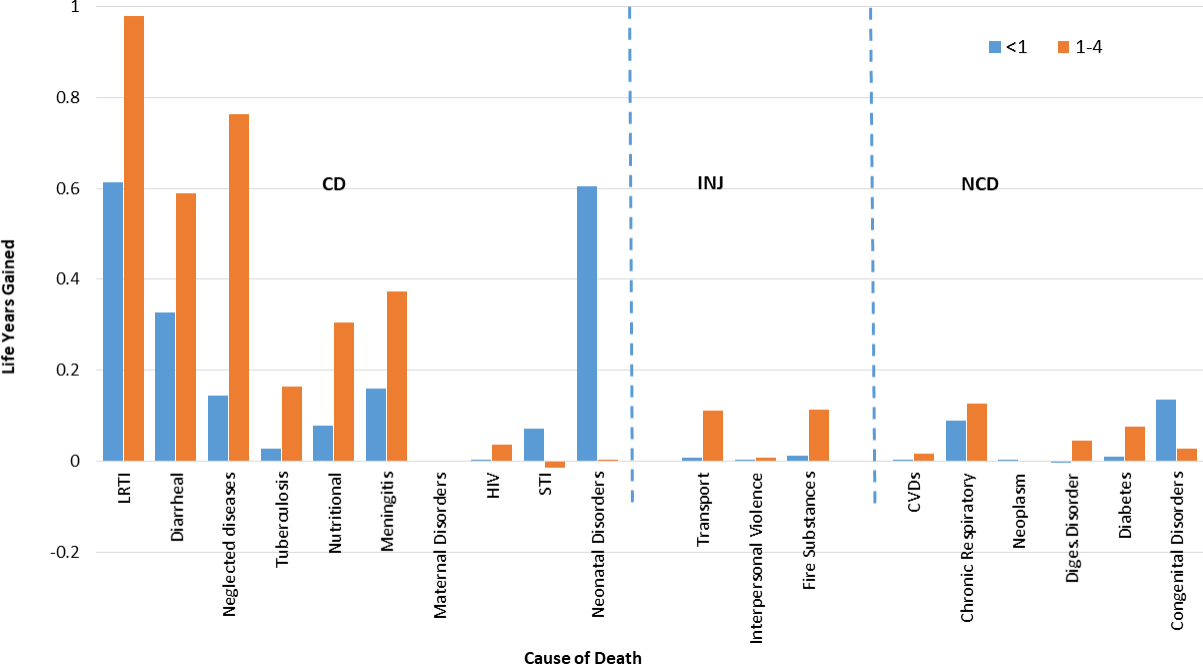
Decomposition of age-cause of gains in life expectancy for infants and under five children between 1990 and 2015. This figure shows the cause-specific attributions in the gains of life expectancy in Ethiopia for infants and under-five children from a reduction in burden of disease. The orange bars indicate the under-five life expectancy improvement and the blue bars indicate the gains in life expectancy for infants attributed to each specific cause of mortality. The values on the horizontal axis indicate the causes of mortality and the values on the y-axis indicate the additional years contributed to life expectancy gain from 1990 to 2015.

Another interesting question is which specific cause of death has really contributed to the improvement in life expectancy in children? Before a general look at the cause specific analysis, decomposition of the under-five mortality by cause and age shows that the major improvements in life expectancy in this group comes from reduction in the burden of lower respiratory infection, diarrheal diseases and neonatal disorder among infants. Altogether, this accounts for more than 47% of the gain in life expectancy in this group and 7.4% among the total population. Similarly, observation of the contributors for improvement in life expectancy for children aged one to five years indicates that a reduction in LRT infection, diarrheal disease, neglected tropical disease, nutritional deficiencies, and meningitis contributed to more than 46.9 percent of the gain in life expectancy for the group and 14.4% for the total population (Fig 3).

**Fig 4:**
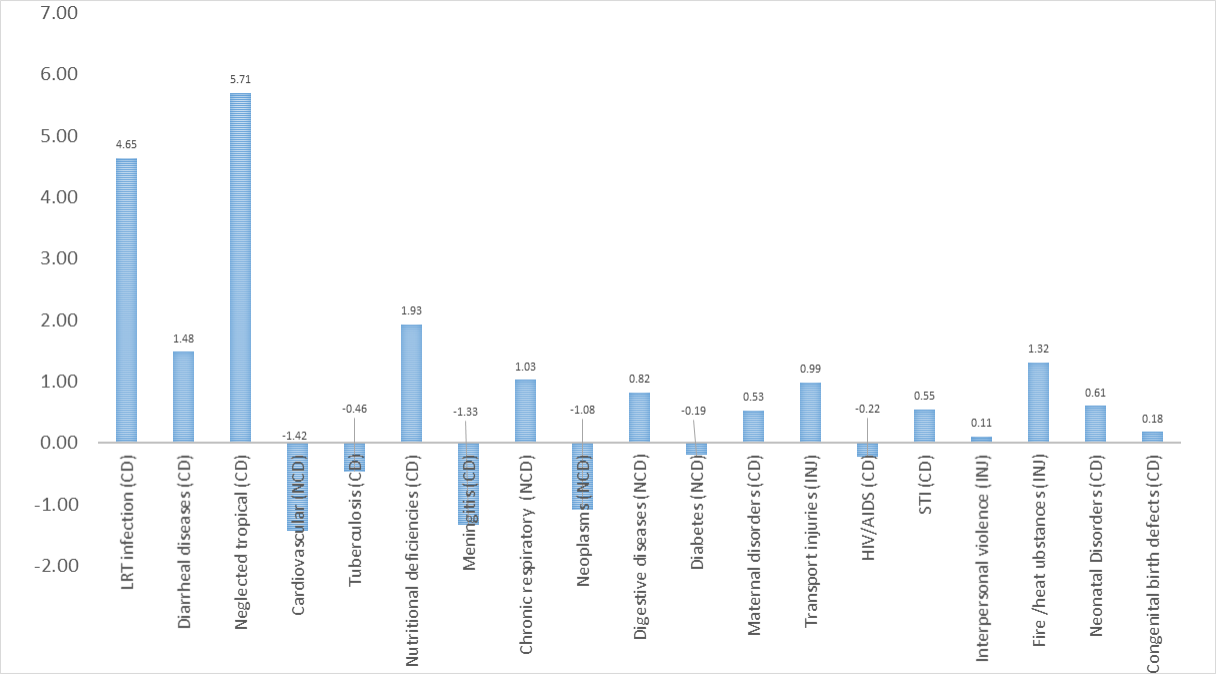
Life expectancy gains apportioned to different causes of death in Ethiopia from 1990 to 2015. This graph shows the average life expectancy that is attributed by either reduction or shift in mortality from specific causes of mortality from 1990 to 2015. The values on the vertical axis represent the number of years attributed to specific cause of mortality.

Fig 4 shows the contribution of specific causes of mortality in the improvement of life expectancy. Reductions in mortality from major communicable disease burdens including neglected tropical diseases, lower respiratory tract infections and diarrheal diseases contributed to an increase in life expectancy by 5.7, 4.6 and 1.5 years respectively from 1990 to 2015. Reduction in nutritional deficiencies have also contributed to 1.9 years of life across the study period. Of 5 non-communicable disease burdens, only chronic respiratory diseases and digestive diseases contributed to life expectancy improvement, whereas diabetes, neoplasm and cardiovascular diseases had negative impacts on life expectancy decline from 1990 and 2015. Consequently, the net impact of the five non-communicable diseases on life expectancy was negative, -0.8, from 1990 to 2015.

## Discussion

The findings of this research substantiate other study findings, which indicate a significant progress in the burden of disease reduction in Ethiopia. For most communicable diseases, both age specific mortality rates and rates of years of life lost saw a reduction from 1990 to 2015. The order of the major causes of burden of diseases based on YLL both in 1990 and 2015 indicates that the top causes of mortality are communicable diseases. However, there is also a significant decline in burden of communicable diseases across time. Lower respiratory tract infection leads by a 16.6% reduction rate of age-standardized years of life lost. Similarly, calculation of the life years gained in 1990 shows that the elimination of LRT infections could have contributed to the gain of 4.2 years followed by diarrheal and neglected tropical diseases at 3.0 and 2.2 years, respectively. These shifts indicate the reduction in premature deaths due to either change in disease epidemiology or improved childhood health promotion as well as disease prevention activities. The finding from lower respiratory infection in Ethiopia might specifically be following the introduction of an expanded immunization strategy in 2007 [25]. Two new vaccines for Haemophilus influenza type B and Hepatitis B were combined with the routine trivalent vaccines (DPT) to form a pentavalent one. This might have contributed to the prevention of respiratory diseases in the country [26].

On the other hand, the major gain in life expectancy from 1990 to 2015 also attributed to neglected tropical diseases by 5.7 years followed by lower respiratory infections (4.7 years) and diarrheal diseases (1.5 years) which is in line with the GBD 2016 report[27,28]. Findings from the GBD 2013 collaborators [29] who found trends of reductions in child deaths from diarrhea, lower respiratory infections, and neonatal causes in low-income regions also support the study result.

Burden of disease study in the Amhara region [30] also showed similar findings where the highest number of DALY lost were obtained for acute respiratory tract infections, malaria, diarrhea, tuberculosis, and maternal and perinatal causes; out of which communicable, maternal and perinatal problems contributed to about 68% the burden. Another study on deaths in private and public hospitals in Addis Ababa by Misganaw et.al [31] also found that 59% of the deaths were attributed to group I (infectious) diseases, 31% to group II (non-communicable) diseases, and 12% to injuries. The proportion of non-communicable diseases and injuries in this study were found to be higher than the Butajira and Amhara rural studies. Measurement of burden of disease in rural areas of Western Kenya [32] and Zimbabwe [33] on the other hand showed a different picture. For Kenya, more than 71.5% of the morbidity and 28.9% of deaths were due to malaria. In Zimbabwe, more than 49% of the burden of disease was due to HIV/AIDS.

The mean years of life lost for the life table population (nLx) in this study at a national level in 1990 and 2010 are 23.0 and 17.9, respectively. This brought to a a consequent fall in entropy from 0.5 to 0.3. Entropy, which is calculated as the ratio of mean YLL for life table population (nLx) to life expectancy at age zero [34]. Entropy has already fallen below 0.2 [34] in most developed countries. A higher entropy means that more people are losing their age prematurely. The above results imply a big fall in years of life lost for the nation in the past 20 years, although the value of entropy is still very big compared to the global average.

Other causes of mortality explained additional 8 years of life years gained in the 1990s, with only a 2.2 years gain in the year 2015. Even though it is not included in the model, an in-depth analysis of other causes of mortality for 1990 showed that war and legal interventions account for a large portion of the burden of diseases in 1990. A study on development consequences of armed conflict by Gates S et.al [35] has a similar finding; where a medium-sized conflict with 2500 battle deaths reduces life expectancy by about 1 year, and increases infant mortality by 10%. Another study by Plümper T et.al [36] and Li Q and Wen M [37] showed that armed conflict tends to decrease the life expectancy of women much more than men. The current finding in our study might indicate the contribution of the peace dividend that the country has enjoyed for the past 25 years towards the improvement in Ethiopian life expectancy. The term peace dividend is coined to indicate the extra money, time, and human resource gained from reduced defense budgets during conflicts that can be invested in various social and economic sectors. Most literatures agree that a cut in military expenditure will give the public sector an opportunity to fund social and other economic sectors [38,39]

On the other hand, decomposition of life expectancy confirms that improvements in both infants and children aged 1–4 contribute nearly half (45.8%) of the life expectancy gained from 1990 to 2015. These findings are also supported by studies from global child mortality analysis [40–42], where a reduction of 2·0–2·4 million child deaths were found at a global level. One of these studies conducted by Li Liu et.al [40] found major reductions in pneumonia, diarrhea, and measles. Despite the slight reduction in burden of diseases among adolescents, youth, and the adult population, the gain in life expectancy from these age groups is not encouraging. This signals the presence of a room for further improvement on the lives of the general population through identification, prioritization, and addressing of the major causes of morbidity and mortality for those groups. The work of Tiamaeus et.al. [43] who showed a rising adult mortality in eastern Africa supports this finding. The improvement in neonatal health and maternal health condition seems very slow, which is evidenced by the small gain in life expectancy. This finding supports the works of Ngoc et.al. [44], which shows a slow progress in neonatal disorders despite child mortality reductions that requires further exploration.

Beyond the need to focus on increasing life expectancy, chronic diseases are seen as emerging causes of mortality. These diseases include cardiovascular disease, neoplasms, and diabetes mellitus. In this regard, in 2015, the elimination of cardiovascular and circulatory disorders could have contributed to a gain of 2.6 years followed by neoplasms (1.8 years). This implies that public health efforts in Ethiopia ought to make non-communicable diseases their focus of interventions to improve the longevity of its citizens. This is supported by overwhelming evidence that shows the growing burden of non-communicable disease in developing countries, calling for a prevention strategic planning through lifestyle improvements and access to better health care services to address non communicable diseases.

In a similar context, mortality and disease burden from interpersonal violence has also increased, which goes in line with the findings in the global burden of disease, which ranks Ethiopia fourteenth in interpersonal violence and twelfth in road traffic related deaths [45]. With all these evidences, it is clear that the country is facing a triple burden of disease from communicable, non-communicable, and transport related problems. However, this study argues that the attention of public health in Ethiopia is mainly inclined towards the prevailing communicable diseases pinpointing a need for a critical public attention. The recognition of NCD’s burdens in Ethiopia Health Sector Transformation Plan (HSTP 2015/16-2019/20) is a very welcome step [46]. However, the performance measures and strategic initiatives for NCDs in the HSTP are much less specified in comparison with communicable diseases. This may pose heavy health burdens by NCDs in the coming years. More specific targets and priorities on the health intervention for NCDs would contribute to not only reducing in health burdens but also creating healthy population to harness the expected demographic dividend in Ethiopia.

In addition, as the country’s productive labor force increases, it might bring increased risks to interpersonal conflicts and violence following challenges in unemployment or underemployment, fast urbanization, and the rising population. This might require cultural, legal, and other forms of measures to be in place to address violence, transport related injuries, and other forms of growing public health problems at an early phase.

## Limitations of the study

This study used a secondary data obtained from IHME, which was estimated using statistical models. While effective civil registration systems remain the ‘gold standard’ source for continuous mortality measurement, less than 25% of deaths are registered in most African countries. Ethiopia, like most of the other African nations, does not have a functional vital registration. Accordingly, the data that was used might lack depth and errors might be introduced during modeling. The study also does not consider uncertainty intervals for its estimation. As a result, this study is not immune to suffer from various shortcomings of secondary data analysis.

## Conclusion

Measurements on the burden of disease in Ethiopia found that communicable diseases including lower respiratory disease, diarrheal disease and neglected tropical diseases are the leading contributors. However, a percentage change in the rate of age adjusted years of life lost shows shifts in the rank and age distribution of these causes of mortality across time. These changes also resulted in an improvement in the life expectancy of the population from 1990 to 2015. This study confirms that nearly half of the improvement in life expectancy is attributed to improvements in under-five health condition, which is promising for a country with high child mortality rate compared to other nations globally. In a similar context, most of these improvements are attributed to reductions in diarrheal diseases, lower respiratory diseases, neglected tropical diseases, and tuberculosis. However, it is also important to note that there is potential for further improvements in the health conditions of the general adult population, which has showed relatively slower improvement in life expectancy. The growing burden of mortality from cardiovascular diseases, diabetes and neoplasms also need a proactive intervention. Similarly, interpersonal violence and traffic accidents seem to be overlooked despite showing an increase in burden overtime, calling for the public’s attention and the development of a strategic plan and interventions to address them.

## Supporting information

S1 Data Causes of mortality data for Ethiopia from 1990 to 2015, retrieved from the Global Burden of Disease Study, IHME. This file contains data from the Global Burden of Disease Study retrieved from the Institute of Health Metrics and Evaluation at http://ghdx.healthdata.org/gbd-results-tool. It has four different sheets; *“data-PLOS”*, *“pivot”*, *“code”* and *survival”*. ***data-PLOS*:** This sheet includes mortality data files of Ethiopia for the years 1990, 2000, 2010 and 2015 directly retrieved from the global burden of disease study; IHME website; ***Pivot*:** This sheet is a pivot format of the data-PLOS which helps for further categorization and ordering the causes of mortality based on their burden in terms of death, sex, year and age categories; ***Code*:** the code sheet is a direct download of the causes of death from IHME code book. It helps to identify each cause by level (Hierarchy I, Hierarchy II, and Hierarchy III);***Survival*:** This sheet includes the final 20 causes of death (including others) that are selected for life expectancy decomposition by year ready to be exported to survival 6-software.
(XLSX)

## Abbreviations

AFI: Acute febrile illnesses
DALY: Disability adjusted life years lost
GBD: Global burden of diseases
HIV/AIDS: Human immune virus/acquired immune deficiency syndrome
ICD: International classification of diseases
IHME: Institute of health metrics and evaluation
LRT: lower respiratory tract
NCD: Non Communicable Diseases
NGTDS: Neglected Tropical Diseases
STI: Sexually transmitted infections
WHO: World health organization
YLD: Years lost due to disability
YLL: years of life lost
